# Food Packaging Materials for One-Dose Packaging for Enhanced Stability of Hygroscopic Medications

**DOI:** 10.3390/ph19010163

**Published:** 2026-01-16

**Authors:** Takayuki Yoshida, Kiyotaka Ushijima, Natsumi Nishimura, Makoto Toda, Miho Morikawa, Kazuhiro Iwasa, Takashi Tomita

**Affiliations:** 1 Laboratory of Practical Community Collaboration, Center for Social Pharmacy, Teikyo Heisei University, 4-21-2 Nakano, Tokyo 164-8530, Japan; 2 Development Department, Maruto Sangyo Co., Ltd., 892-1, Hikata, Ogori 838-0112, Japan; k-ushijima@marutosangyo.co.jp (K.U.); n-nishimura@marutosangyo.co.jp (N.N.); 3 Toshiba Nano-Analysis Corporation, 1 Komukai Toshiba-Cho, Kawasaki 212-8583, Japan; makoto.toda@nanoanalysis.co.jp (M.T.); miho.morikawa@nanoanalysis.co.jp (M.M.); kazuhiro.iwasa@nanoanalysis.co.jp (K.I.); 4 Department of Pharmaceutical Sciences, School of Pharmacy, International University of Health and Welfare, Narita Campus, 4-3 Kozunomori, Narita 286-8686, Japan; 5 Department of Pharmacy, International University of Health and Welfare Mita Hospital, 1-4-3 Mita, Tokyo 108-8329, Japan

**Keywords:** one-dose packaging, hygroscopic medications, moisture barrier, food packaging materials, pharmaceutical stability, medication adherence

## Abstract

**Background/Objectives:** One-dose packaging is beneficial for older adults and those on multiple medications because it ensures that no doses are missed and supports medication adherence. However, conventional one-dose packaging materials have high moisture permeability, making them unsuitable for the storage of hygroscopic medications. We evaluated the barrier performance of food packaging materials against moisture and oxygen and investigated their potential to enhance the physical stability of the highly hygroscopic sodium valproate, under stressed storage conditions. **Methods:** Barrier performance was evaluated by measuring the water vapor transmission (WVTR) and oxygen transmission rates of each packaging material. Then, we evaluated the stability of sodium valproate tablets in different food packaging films by measuring weight change, breaking force, and visual appearance over 14 days under stressed storage conditions (35 °C and 75% relative humidity). Conventional cellophane-laminated polyethylene was used as the reference. **Results:** The WVTR of the food packaging films were below 2 g/m^2^/day, less than that of the conventional material. Tablets stored in Materials A and B showed weight increases of no more than 1.2% after 3 days, whereas the maximum increase among all food films was 3.7% (Material C). For Materials A and B, the breaking force remained measurable and the visual appearance unchanged throughout the 14-day period, whereas Material C became unmeasurable by day 14. Tablets packaged in cellophane-laminated polyethylene exhibited deliquescence, with visible deformation and stickiness within 3 days, rendering them unmeasurable. **Conclusions:** Food packaging materials with high barrier performance offer a practical, safe, and effective solution for one-dose packaging of hygroscopic medications, potentially expanding their clinical use and improving adherence.

## 1. Introduction

One-dose packaging is a dispensing method in which medications are packaged in a single sachet organized according to their administration schedule [1]. This method is commonly used for older adult patients and individuals on multiple medications, as it helps prevent missed doses and improves medication adherence [2,3,4]. The one-dose packaging materials used for this purpose are restricted to proprietary products supplied by the packaging machine manufacturers due to structural constraints, effectively limiting the available options to cellophane-laminated polyethylene or glassine. Unlike press-through packages (PTPs) or strip-packaged products, these materials exhibit high moisture permeability, making them unsuitable for the storage of hygroscopic medications [5]. Consequently, pharmacists often dispense such medications in PTP sheets or strip packages instead of one-dose packages [6]. However, the inability to include hygroscopic medications in one-dose packaging is of clinical concern [7]. Previous surveys showed that pharmacists are eager to incorporate such medications in one-dose packaging whenever practicable and express a strong need for improved barrier performance of packaging materials [8].

To address these challenges, adapting food packaging paper—widely used globally for its stable supply and cost-conscious design—to one-dose packaging may offer a realistic solution, potentially providing practical benefits for patients and pharmacists by facilitating accurate, adherence-supporting dispensing. Food packaging materials are designed to offer high-barrier properties against moisture and oxygen to preserve food quality and shelf-life. Furthermore, multilayer films exhibiting these functions are used in the food industry [9,10]. Hence, using these food packaging materials for one-dose packaging can make medications less susceptible to external environmental factors and potentially improve the stability of hygroscopic medications. Although the expansion of one-dose packaging can improve medication adherence, it also increases the use of packaging materials, raising sustainability concerns regarding resource consumption, waste generation, and environmental impacts associated with pharmaceutical packaging [11,12]. Therefore, balancing patient benefits with environmental considerations is essential when developing new packaging materials.

The 18th edition of the Japanese Pharmacopoeia [13], the European Pharmacopoeia, [14] and the United States Pharmacopeia [15] specify quality assurance tests, such as weight variation and tablet hardness, for assessing pharmaceutical stability, both of which can be affected by moisture uptake [8,16]. Deterioration of medication quality may result not only in failure to meet pharmacopeial standards but also in reduced therapeutic efficacy. Therefore, maintaining the stability of medications in one-dose packaging is essential. For example, studies evaluating the stability of hygroscopic medications in one-dose packaging have shown that Glucobay tablets can be stably stored in polyethylene bags with desiccants under daily environmental conditions [17]. Belsomra tablets maintain their quality when stored in a refrigerator [18], and specially designed moisture-suppression bags can minimize changes in weight and breaking force under stressed conditions [8,16]. While such studies have focused on optimizing storage conditions, no studies have directly evaluated the effect of barrier properties of one-dose packaging materials on the medication stability.

In this study, we aimed to evaluate the barrier performance of food packaging materials against moisture and oxygen and investigate their potential use in one-dose packaging to enhance the physical stability of hygroscopic medications under stressed storage conditions. Although potassium L-aspartate tablets exhibited the highest incidence of moisture-related issues in a previous clinical survey [8], they could not be selected for the present study due to supply shortages and distribution instability [19,20]. In the same survey, sodium valproate tablets were reported as the second most frequently affected medication, supporting their clinical relevance and justifying their selection for this study. Consequently, sodium valproate, which showed the second-highest incidence, was selected as the target medication. While sodium valproate is chemically relatively stable, it is notoriously susceptible to rapid physical liquefaction upon moisture exposure [21]. Therefore, we prioritized evaluating the physical stability of this medication as a primary step to demonstrate the protective potential of high-barrier food packaging materials under stressed storage conditions (35 °C and 75% relative humidity).

## 2. Results

### 2.1. Choice of Packaging Materials

Three types of packaging materials used for food preservation were selected (Materials A, B, and C), and the one-dose packaging paper was selected based on its prevalence in pharmacy practice.

### 2.2. Choice of Test Medication

To ensure applicability in clinical settings, target medications were selected based on their hygroscopicity issues in clinical practice, as noted in a previous survey [8].

### 2.3. Barrier Performance Evaluation

**Figure 1:**
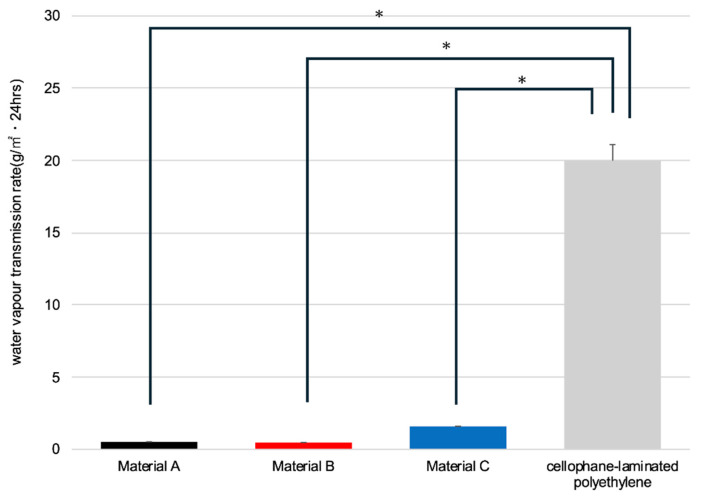
Comparison of the water vapor transmission rate per material. Measurements were performed in quintuplicate (*n* = 5). Error bars indicate standard error of the mean (SEM). * *p* < 0.0001 for all comparisons (Tukey–Kramer test).

The mean ± standard error of the water vapor transmission rates (WVTRs; g/m^2^·24 h) were as follows: Material A, 0.52 ± 0.02; Material B, 0.48 ± 0.01; Material C, 1.59 ± 0.02; and cellophane-laminated polyethylene, 19.99 ± 1.12. Significant differences in the WVTR were observed among the materials [F(3, 16) = 290.45, *p* < 0.0001]. Post hoc Tukey–Kramer tests revealed significant differences between the cellophane-laminated polyethylene and all food packaging materials (*p* < 0.0001), whereas no significant differences were observed among Materials A, B, and C (*p* > 0.05, Figure 1).

The oxygen transmission rates (OTRs; cc/m^2^·24 h·atm, 23 °C wet) were 0.10, 0.26, and 2.76 for Materials A, B, and C, respectively. OTR of the cellophane-laminated polyethylene could not be evaluated because it exceeded the measurement upper limit (2000, Figure 2).

### 2.4. Changes in Tablet Weight

The weight change rate (%) on day 3 was 0.97 ± 0.02, 1.18 ± 0.02, and 3.69 ± 0.04 for Materials A, B, and C, respectively. The weight change continued to increase gradually; on day 7, the values were 1.89 ± 0.03% for Material A, 2.59 ± 0.05% for Material B, and 7.66 ± 0.23% for Material C. By day 14, Materials A and B showed weight increases of 3.51 ± 0.07% and 4.40 ± 0.11%, respectively, resulting in a difference of approximately 0.9% between these two high-barrier materials. Owing to tablet deliquescence, tablets in the cellophane-laminated polyethylene could not be evaluated on day 3, and Material C was unmeasurable by day 14. Overall, tablet weights showed a consistent increase over the course of the study for all materials. All values are presented as the mean ± standard error of the mean (SEM).

**Figure 3:**
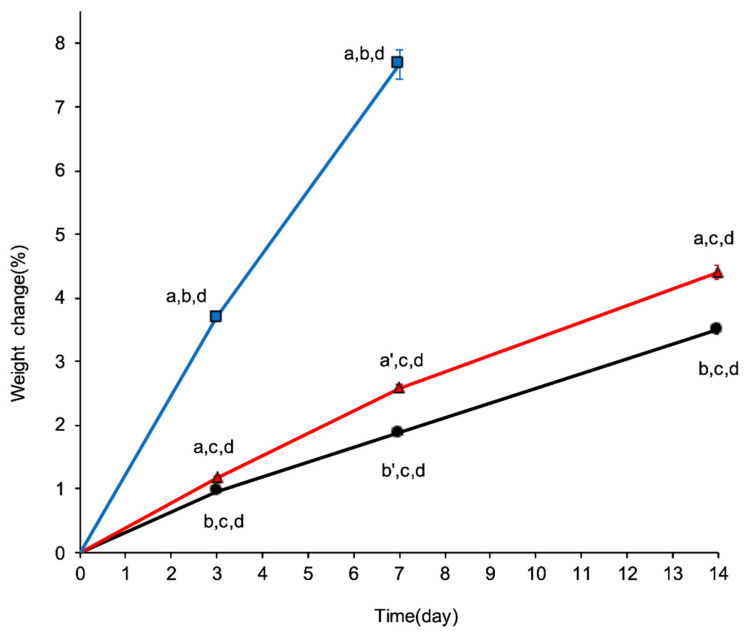
Changes in tablet weight (%) over time when stored in different packaging materials under stressed conditions (35 °C and 75% relative humidity). ● (black), ▲ (red), and 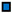 (blue) represent Materials A, B, and C, respectively. Measurements were performed on days 3, 7, and 14 (*n* = 5). Error bars represent the standard error of the mean (SEM). Cellophane-laminated polyethylene could not be evaluated because of its inability to maintain tablet integrity. a, b, and c indicate statistically significant differences compared to materials A, B, and C, respectively (a *p* < 0.001, a’ *p* < 0.01, b *p* < 0.001, b’ *p* < 0.01, and c *p* < 0.001; Tukey–Kramer test); d indicates a significant difference from day 0 (*p* < 0.001, Tukey–Kramer test).

One-way analysis of variance (ANOVA) revealed significant differences in weight change over time for each material: Material A, F(3, 16) = 1339.55, *p* < 0.0001; Material B, F(3, 16) = 898.10, *p* < 0.0001; and Material C, F(2, 12) = 811.89, *p* < 0.0001. Tukey’s Honest Significant Difference tests showed significant differences from baseline (day 0) to day 3 for all materials (*p* < 0.0001), indicating a significant increase in weight due to moisture absorption. Additionally, the ANOVA at each time point revealed significant differences between the materials: Day 3, F(2, 12) = 2733.05, *p* < 0.0001; Day 7, F(2, 12) = 528.30, *p* < 0.0001; Day 14, F(1, 8) = 45.21, *p* < 0.0001. Differences were observed between the materials across all time points (Figure 3).

### 2.5. Changes in Tablet Breaking Force

**Figure 4:**
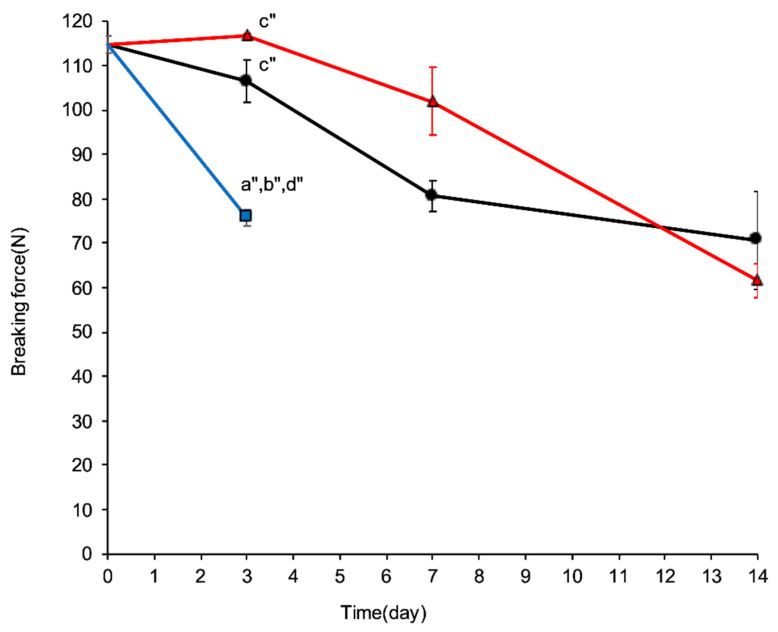
Changes in tablet breaking force over time when stored in different packaging materials under stressed conditions (35 °C and 75 % relative humidity). ● (black), ▲ (red), and 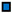 (blue) represent Materials A, B, and C, respectively. Measurements were performed on days 0, 3, 7, and 14 (*n* = 5). Error bars represent the standard error of the mean (SEM). Cellophane-laminated polyethylene could not be evaluated because of its inability to maintain tablet integrity. a, b, and c indicate statistically significant differences compared with Materials A, B, and C, respectively (a″, *p* < 0.05; b″, *p* < 0.05; c″, *p* < 0.05; Steel–Dwass test); d indicates a significant difference from day 0 (d″, *p* < 0.05, Steel–Dwass test).

The initial average tablet breaking force was 114.74 ± 1.96 N. On day 3, the average tablet breaking forces were 106.5 ± 4.79 N, 116.70 ± 0.44 N, and 75.71 ± 1.82 N for Materials A, B, and C, respectively. Tablets in the cellophane-laminated polyethylene and Material C could not be evaluated on day 14 because of deliquescence. Kruskal–Wallis tests revealed significant changes in tablet hardness over time: Material A, H(3) = 13.12, *p* = 0.0044; Material B, H(3) = 11.86, *p* = 0.0079; and Material C, H(1) = 7.08, *p* = 0.0078. The Steel–Dwass tests showed significant differences in tablet hardness between days 0 and 3 for Material C. When comparing the materials at each time point, a significant difference was found only on day 3 [H(2) = 10.69, *p* = 0.0048]. No significant differences were observed on days 7 [H(1) = 3.15, *p* = 0.0947] and 14 [H(1) = 0.01, *p* = 0.9163] (Figure 4).

### 2.6. Visual Appearance

**Figure 5:**
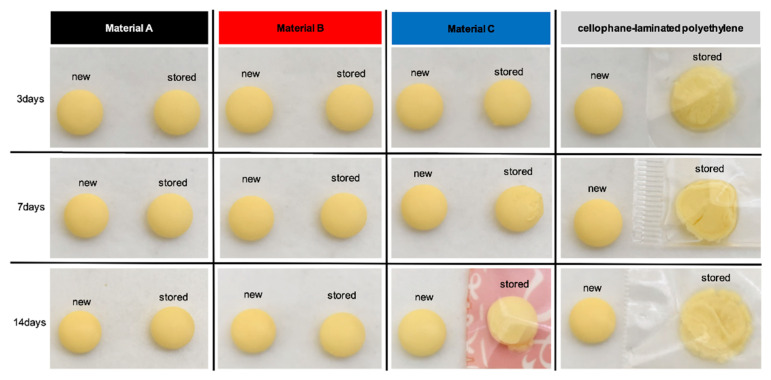
Representative photographs of sodium valproate tablets stored in one-dose packages made of Materials A, B, C, and cellophane-laminated polyethylene under stressed conditions (35 °C and 75% RH) for 3, 7, and 14 days. Each panel shows tablets before (new) and after (stored) storage. The headers for Materials A, B, and C are color-coded in black, red, and blue, respectively.

Tablets stored in cellophane-laminated polyethylene showed deliquescence by day 3. Tablets in Material C showed slight deliquescence on day 3, which became more pronounced by day 7. Tablets in Materials A and B maintained their visual integrity through day 14 (Figure 5). Deliquescence is clearly visible in Figure 5; therefore, instrumental color measurements were deemed unnecessary for this analysis.

## 3. Discussion

In this study, we demonstrated that food-grade high-barrier packaging materials can substantially improve the physical stability of hygroscopic pharmaceutical tablets when applied to one-dose packaging. While previous studies have focused primarily on storage conditions or auxiliary measures such as desiccants, the present findings highlight the critical role of intrinsic barrier properties of packaging materials in mitigating moisture-induced deterioration.

Packaging is recognized as a critical component of pharmaceutical stability, as it directly controls exposure to environmental factors such as moisture, oxygen, and light, which can significantly affect the physical and chemical integrity of solid dosage forms [22]. Compared to the conventional one-dose packaging paper (cellophane-laminated polyethylene), food packaging materials exhibited superior moisture and oxygen barrier properties, maintaining tablet shape despite a slight decrease in hardness over 14 days under stressed storage conditions (35 °C and 75% relative humidity). The differences in WVTR among Materials A, B, and C were small compared to those in cellophane-laminated polyethylene; however, the WVTR of Material C was approximately three times higher than that of Materials A and B, and the weight change rate similarly showed a threefold increase. Materials A and B feature a transparent vapor-deposited inorganic layer on 12-μm PET, forming a dense physical barrier, whereas Material C uses a polymeric coating, polyvinylidene chloride (PVDC), as its barrier layer. The vapor-deposited inorganic layer provides fewer diffusion pathways for water vapor at the molecular level, which likely accounts for the approximately threefold higher WVTR observed in Material C compared with Materials A and B [23]. This finding suggests that the weight change rate strongly depended on the WVTR of the packaging material. Although Materials A and B had the same barrier layer structure and similar oxygen and moisture permeabilities, a maximum difference of 0.9% in weight change was observed on day 14. This subtle variation may be attributed to microscopic defects or pinholes in the inorganic vapor-deposited layer that can arise during the lamination or heat-sealing process, as such defects have been reported to increase water vapor permeation in inorganic barrier coatings [24]. This difference did not affect tablet hardness or visual appearance and was considered clinically negligible. Potential contributing factors include differences in material thickness, structural uniformity, and minor variations in sealing conditions, including the difference between manual and automated fabrication. However, these factors are not expected to hinder practical applications or alter the overall conclusions. These results suggest that WVTR can serve as a useful indicator for evaluating the physical stability of hygroscopic pharmaceuticals in one-dose packaging.

In addition, oxygen permeability showed apparent differences among the materials; however, the OTR values obtained in this study were based on single measurements (*n* = 1) and should therefore be interpreted as descriptive observations rather than for comparative or statistical inference. While the OTR of cellophane-laminated polyethylene exceeded the measurement limit, the food-grade packaging materials exhibited lower oxygen permeation under the tested conditions. Although these findings tentatively suggest a potential contribution of oxygen barrier properties to improved tablet stability, definitive conclusions regarding oxygen-related effects cannot be drawn from the present data. Although oxidative degradation was not directly evaluated in this study, oxygen is a well-recognized factor affecting pharmaceutical product stability [22]. Future studies incorporating replicated OTR measurements and chemical stability assessments are necessary to clarify the role of the oxygen barrier performance in one-dose packaging systems.

Sodium valproate is a well-established hygroscopic and deliquescent powder [21]. In this study, the suppression of deliquescence indicates the mitigation of moisture-driven physical degradation of the active pharmaceutical ingredient. Although no chemical assay was performed, maintaining physical integrity under high-humidity conditions is considered a prerequisite for chemical stability, and these findings may imply relative preservation of the chemical stability when food-grade packaging materials are used. In addition, although the suppression of moisture uptake contributes to improved stability, the dissolution behavior of hygroscopic drug products remains sensitive to moisture exposure [25] and should be carefully considered when evaluating overall drug performance.

This study suggests that high-permeability materials, such as cellophane-laminated polyethylene, may create conditions similar to those of open storage. Indeed, studies have reported quality deterioration of medications with insufficient stability information when stored in unsealed environments [26]. Thus, the results of this study may be applicable to other medications, highlighting the risks associated with the use of low-barrier packaging for one-dose dispensing. Inadequate barrier materials may pose the same risks as unwrapped storage, highlighting a previously overlooked issue. In Japan, immediate-release tablet formulations are generally considered unsuitable for one-dose packaging because of the high hygroscopicity of sodium valproate, making sustained-release formulations a frequently used alternative. Although these tablets are generally perceived as more stable, sustained-release formulations do not guarantee sufficient stability. In a study by Redmayne et al., repackaging sustained-release sodium valproate tablets into dose administration aids (DAAs) led to deliquescence and coating degradation under high temperature and humidity conditions in Australia [27]. Similarly, repackaging telmisartan tablets into DAAs resulted in significant formulation-dependent variability in stability [28]. These findings imply that the dosage form alone does not ensure stability, and that the barrier performance of the packaging material is crucial. Although this study focused on immediate-release tablets, the insights gained may also be applied to sustained-release tablets and other formulations.

Using high-barrier materials may enable stability and minimize the need for formulation changes. Furthermore, one-dose packaging often involves co-packaging of multiple drugs. Hence, storing together hygroscopic and other type of tablets may improve their stability [29]. Increasing the number of tablets in a package can suppress the increase in internal humidity [30]. Evaluating these effects under real-world dispensing conditions may further support the stabilization outcomes reported in this study.

Pharmacists are unable to assess barrier performance based on the appearance of the packaging paper, thereby hindering accurate evaluation of the impact of material differences on medication stability. The food packaging materials used in this study not only met the functional requirements of the pharmaceutical practice, including transparency, heat sealability, ease of manual cutting, safety, and availability, but also demonstrated excellent performance in preserving tablet stability. The clinical adoption of these materials may facilitate the broader application of one-dose packaging for hygroscopic medications, thereby improving medication adherence and continuity of care.

Nonetheless, broader adoption of one-dose packaging may increase the use of packaging materials and sustainability concerns. Conventional and food packaging films impose environmental burdens during production and disposal. However, the food packaging materials evaluated in this study demonstrated improved stability for hygroscopic tablets, which may help reduce the risk of medication deterioration, wastage, and re-dispensing. Transparent vapor-deposited PET films are widely used in food packaging and have demonstrated high barrier performance in various non-pharmaceutical applications, particularly for moisture and oxygen control [31]. Although the present study did not include a formal economic evaluation, the potential clinical implications, such as reducing medication wastage and supporting stable dispensing workflows, should be considered alongside sustainability issues when evaluating future implementation.

This study has some limitations. First, only a limited number of materials and medications were tested, and pharmacokinetic or therapeutic evaluations were not performed. Second, although chemical stability is crucial for understanding overall pharmaceutical stability, it was not assessed; therefore, future research should include these analyses. Third, modeling tools were beyond the scope of this study, but such approaches, which consider factors such as sorption isotherms of the drug, WVTR of the packaging material, and long-term storage conditions, can complement experimental findings by predicting in-package humidity and long-term stability. Finally, future studies should investigate the use of high-barrier materials in multi-medication one-dose packaging, other dosage forms, and real-world clinical environments. Despite these limitations, the present study provides fundamental and practical insights into how packaging materials affect the stability of hygroscopic medications, offering a valuable foundation for their selection in pharmaceutical practice.

## 4. Materials and Methods

### 4.1. Packaging Material Selection

The selection pool initially included several commercially available high-barrier films widely used in the food industry. To identify candidates suitable for pharmaceutical one-dose packaging, a screening process was conducted based on consultations with food packaging engineers and clinical pharmacists. Based on discussions with professionals working on a food packaging material manufacture company and pharmacists with clinical experience, the following criteria were used to select the candidate materials: currently used in food packaging, high moisture-barrier performance, easy to procure, heat-sealable, transparent, and cuttable by hand. Three food packaging materials that met all the criteria were selected.

Material A consists of a 12-μm polyethylene terephthalate (PET)/12-μm transparent vapor-deposited PET/40-μm linear low-density polyethylene (LLDPE) used for products such as furikake and dried and health foods. Material B has the same PET layers but uses 60-μm LLDPE and is also used for the same type of products as Material A. Material C, used for pickled foods, is composed of 20-μm polyvinylidene chloride (PVDC)-coated oriented polypropylene (OPP) film/20-μm low-density polyethylene (LDPE)/40-μm LLDPE.

Based on past surveys showing that cellophane-laminated polyethylene (Cello Poly) materials are commonly used, we selected Cello Poly 20 μm (Yuyama Co., Ltd., Osaka, Japan) as the one-dose packaging paper of reference [8] (Table 1).

### 4.2. Pharmaceutical Product Tested

Sodium valproate tablets (Depakene 200 mg; LOT 971ABK; Kyowa Kirin Co., Ltd., Tokyo, Japan) were used as the test pharmaceutical product in this study. The tablets had a diameter of 9.2 mm and a thickness of 4.9 mm, with a mean mass of 0.26 g. The commercial product is supplied in press-through packaging (PTP), with each box containing 100 tablets. According to the package insert, sodium valproate tablets (Depakene 200 mg) contain the following excipients: ethylcellulose, yellow ferric oxide, carnauba wax, croscarmellose calcium, glycerin fatty acid esters, titanium dioxide, magnesium stearate, hydroxypropyl cellulose, hypromellose (substitution type 2910), and D-mannitol. No quantitative chemical assay of drug content or dissolution testing was performed in the present study, as the objective was to evaluate physical stability associated with moisture exposure under different packaging conditions.

### 4.3. Measurement of the Barrier Properties of Packaging Materials

The WVTR and OTR were measured to evaluate the impact of packaging on medication stability. WVTR determination was based on the gravimetric cup method described in JIS Z 0208, which is a standard method for evaluating the water vapor transmission rate of packaging materials under controlled temperature and humidity conditions [32]. In industrial practice, an adapted pouch method, which is a modification of the conventional cup method, is often used for films with relatively high barrier performance. In the present study, this adapted pouch method was applied to all tested materials to ensure consistency across measurements. For the pouch method measurement, each packaging material was sealed to form small pouches (5 mm sealed edges) with a surface area of 20,000 mm^2^, and 20 g of dehydrated calcium chloride was enclosed in the package. The pouches were stored at 40 °C and 90% relative humidity for 8 days. The weight change was recorded, and WVTR (g/m^2^·24 h) was calculated (*n* = 5). A constant-temperature and -humidity chamber (KCL-2000A; Tokyo Rikakikai Co., Ltd., Tokyo, Japan) was used. The OTR was measured using an OX-TRAN (Hitachi High-Tech Co., Ltd., Tokyo, Japan) with a setting configured for wet conditions (*n* = 1). Tests were conducted in an ISO9001-certified factory based on QMS documents [33].

### 4.4. Fabrication of One-Dose Packaging Bags and Insertion of Evaluation Tablets

**Figure 6:**
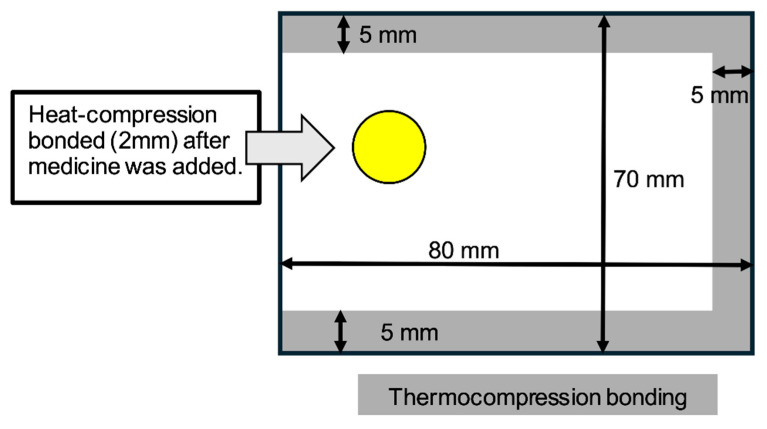
Material processing for one-dose packaging through thermocompression bonding. The yellow circle represents a tablet, and the gray-shaded areas indicate the regions sealed by thermocompression bonding.

Empty pouches of 80 mm × 70 mm were created using a Litrea II one-dose packaging machine (Yuyama Co., Ltd., Osaka, Japan), with 5 mm seal margins. For food packaging materials, bags of the same size were manually fabricated by sealing three edges with a 5 mm width impulse heat sealer (FR-450-5; Fuji Impulse Co., Ltd., Osaka, Japan). The heating and cooling timers were optimized for each material, typically set to level 5 or higher, to ensure a consistent and secure seal, as a one-dose packaging machine could not be used for these materials. To insert the evaluation tablets, one side of the one-dose packaging paper was cut with scissors, the tablet was inserted, and the opening was sealed with a 2 mm heat sealer. For food packaging materials, the tablets were inserted from the open side and then sealed using the same 2 mm heat sealer (Figure 6). The pouches were fabricated and evaluated at different locations. The facility performing the tablet evaluation only had access to a 2 mm heat sealer; thus, all post-insertion seals were uniformly made using this device. Although manual sealing differs from the automated process used by packaging machines, the integrity of each seal was meticulously confirmed through visual inspection and tactile assessment to ensure the absence of leaks or defects.

### 4.5. Evaluation of the Quality of Hygroscopic Medications

The weight of the tablets was measured to establish the baseline value (day 0). Then, tablets were immediately sealed into their respective packaging material and stored under high-temperature and high-humidity stressed conditions [35 °C and 75% relative humidity (RH)] using a constant-temperature and -humidity chamber (PL-3KT; ESPEC Co., Ltd., Osaka, Japan). These conditions were selected instead of standard long-term storage conditions (25 °C/60% RH) to effectively evaluate the barrier performance of the packaging materials within a 14-day observation period. Furthermore, 35 °C/75% RH was chosen to simulate the “worst-case” indoor storage environment in hot and humid climates, such as the Japanese summer, providing a more pragmatic stress test than the official 40 °C accelerated aging protocol. On days 3, 7, and 14, five pouches were removed for each condition, and the weight of the tablets was measured to calculate the percentage change from baseline. After the weighing process, the breaking force changes were tested on the same tablets with a Kiya-type tablet breaking force tester (Fujiwara Manufacturing Co., Ltd., Tokyo, Japan). The breaking force was initially recorded in kgf and then converted to Newtons (N) using an SI conversion factor of 1 kgf = 9.80665 N. The tablets were placed vertically on a test plate for measurement. The experimental period was set at 14 days because home medical care visits are often made twice a month [34].

### 4.6. Evaluation of the Visual Appearance

The tablets stored in each packaging material were photographed on days 3, 7, and 14. Photographs were captured in the same room with a consistent background, placing new (unexposed) tablets alongside for comparison.

### 4.7. Statistical Analysis

WVTR, weight change rate, and tablet breaking force results are presented as the mean ± standard error, and Shapiro–Wilk tests were used to confirm the normality of each of these variables. The results indicated a normal distribution for the WVTR and weight change results (*p* > 0.05), justifying the use of one-way ANOVA followed by Tukey–Kramer post hoc tests for comparisons. For weight change and breaking force, comparisons were made within each material over time (day 0 vs. days 3, 7, and 14) and between different materials on each day (days 3, 7, and 14). The *n*-value for each statistical analysis was 5. The breaking force data did not meet the assumption of normality for all groups (e.g., day 0, *p* = 0.046; material C on day 3, *p* = 0.023); thus, Kruskal–Wallis tests followed by Steel–Dwass tests were used. The *n*-value for each breaking force analysis was also 5. A significance level of α = 0.05 was adopted for all tests, and all tests were two-tailed. All statistical analyses were performed using JMP Pro 18.1.0 software (SAS Institute Inc., Cary, NC, USA). At each time point, five independent pouches containing different tablets were removed and evaluated; therefore, measurements across time points were independent.

## 5. Conclusions

This study demonstrates that enhancing the barrier performance of one-dose packaging materials improves the stability of hygroscopic medications. Particularly, food packaging materials exhibit superior barrier properties compared with those of the conventional cellophane-laminated polyethylene, offering an effective mean for maintaining pharmaceutical quality even under stressed storage conditions. Among the tested films, two food packaging materials maintained tablet integrity for 14 days with weight changes below 1.2%, whereas the conventional material failed within 3 days. These findings suggest that food packaging films are practical and effective alternatives for one-dose packaging, potentially enabling broader applications and improving medication adherence and therapeutic outcomes. Future research should focus on the development of new high-barrier materials and conduct long-term stability evaluations in clinical settings.

In conclusion, the findings of this study provide valuable insights into the development of more effective one-dose packaging solutions that support medication adherence and improve therapeutic outcomes.

## Figures and Tables

**Figure 2:**
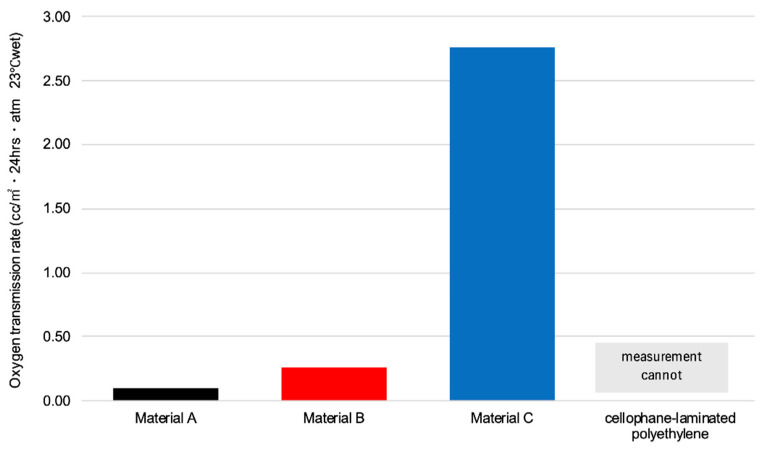
Oxygen transmission rate for Materials A, B, C, and cellophane-laminated polyethylene (*n* = 1). The cellophane-laminated polyethylene could not be measured as it exceeded the upper limit of measurement.

**Table 1:** Composition and intended use of packaging materials analyzed in this study.

Material	Intended Use	Material Composition
Material A	Furikake, dried foods, health foods, etc.	12-μm PET 12-μm transparent vapor-deposited PET 40-μm LLDPE
Material B	Furikake, dried foods, health foods, etc.	12-μm PET 12-μm transparent vapor-deposited PET 60-μm LLDPE
Material C	Pickles	20-μm PVDC-coated OPP film 20-μm LDPE 40-μm LLDPE
Cello poly	One-dose packaging of medications	20-μm cellophane-laminated polyethylene

## Data Availability

The original contributions presented in this study are included in the article. Further inquiries can be directed to the corresponding author.

## Abbreviations

The following abbreviations are used in this manuscript:

PTP	Press-through package
PET	Polyethylene terephthalate
LLDPE	Linear low-density polyethylene
PVDC	Polyvinylidene chloride
OPP	Oriented polypropylene
WVTR	Water vapor transmission rate
OTR	Oxygen transmission rate
DAA	Dose administration aid
